# Simplified MIPI-B prognostic stratification method can predict the outcome well—retrospective analysis of clinical characteristics and management of newly-diagnosed mantle cell lymphoma patients from China

**DOI:** 10.1097/MD.0000000000013741

**Published:** 2019-01-04

**Authors:** Jing-Song He, Xi Chen, Guo-Qing Wei, Jie Sun, Wei-Yan Zheng, Ji-Min Shi, Wen-Jun Wu, Yi Zhao, Gao-Feng Zheng, He Huang, Zhen Cai

**Affiliations:** a The Bone Marrow Transplantation Center & Multiple Myeloma Treatment Center, The First Affiliated Hospital of Medical College, Zhejiang University; b Lymphoma Department, Zhejiang Cancer Hospital, Hangzhou, Zhejiang, China.

**Keywords:** mantle cell lymphoma, MIPI, prognosis, rituximab, treatment

## Abstract

Mantle cell lymphoma (MCL) is an invasive B-cell lymphoma with significant individual differences. Currently, MCL international prognostic index (MIPI) score and tumor cell proliferation index Ki-67 have been proved to be the most important prognostic factors. But the prognostic effect of these factors in Asian population is uncertain. This study aimed to analyze the disease characteristics and prognostic factors of Chinese MCL patients.

A total of 83 cases of newly-diagnosed MCL patients diagnosed by the Department of Pathology of our hospital between January 1, 2011, and May 31, 2016, were enrolled. The disease characteristics, treatment effects, and outcomes of the patients were collected and analyzed.

According to our analysis, MCL cases accounted for 6.2% of non-Hodgkin lymphoma (NHL) cases and mainly occurred in elderly males. But the proportion of patients at stage IV by Ann Arbor staging system and high-risk group by simplified-MIPI (s-MIPI) were significantly lower than that among European patients. Immunochemotherapy containing rituximab was significantly more effective than chemotherapy (overall response rate, [ORR]: 88.5% vs 65.2%, *P* = .021) and significantly prolonged patient survival (progression free survival [PFS]: 45.5 m vs 16.2 m, *P* = .001; overall survival [OS]: 58.3 m vs 22.8 m, *P* = .001). The multivariate analysis showed that the B symptoms, s-MIPI and administration of immunochemotherapy were independent prognostic factors that affected PFS and OS of the patients. s-MIPI and B symptom make up s-MIPI-B stratification method, by which patients in low-risk group of s-MIPI without B symptom were classified as low-risk, patients in high-risk group of s-MIPI and patients in low-risk group of s-MIPI with B symptom as high-risk, the rest as middle-risk. 3-year PFS of the 3 groups were 74.9%, 43.4% and 16.1%, respectively (*P* = .001). 3-year OS were 84.4%, 62.2%, 27.6% (*P* <.001).

Chinese MCL was male predominance. We have a minor proportion of late-stage and high-risk patients compared to European patients. Immunochemotherapy was proved to significantly improve the prognosis of MCL patients. B symptoms, s-MIPI, and administration of rituximab independently influenced the outcome. s-MIPI-B prognostic stratification method may better predict the prognosis of Asian MCL patients. Still, further confirmation in larger populations is needed.

## 1 Introduction

Mantle cell lymphoma (MCL) is a relatively rare B-cell non-Hodgkin lymphoma (NHL) with distinctive clinical, biological and molecular characteristics, which accounts for approximately 3% to 6% of all NHL. MCL cells derive from peripheral B-cells of the inner mantle zone, mostly of naïve pre-germinal center type. t(11;14)(q13;q32) is a characteristic cytogenetic abnormality in MCL patients which leads to structural overexpression of Cyclin D1 (CCND1) on chromosome 11.^[1]^ In recent years, rituximab-based immunochemotherapy, high-dose cytarabine-based intensive chemotherapy as well as autologous stem cell transplantation for young and eligible patients have been to proved to improve the outcome of MCL patients.^[2–4]^ However, MCL is an invasive disease with poor prognosis. The median overall survival (OS) is only 3 to 5 years.^[1,5]^ To our knowledge, there are few data on the treatment and outcome of MCL in real-world China. This study retrospectively analyzed the clinical data, treatment protocols and survival of newly-diagnosed MCL patients admitted to our hospital in recent 5 years, examined the clinical factor relevant to the prognosis and compared the different clinical features with data from western countries.

## 2 Patients and methods

### 2.1 Patients

Continuous newly-diagnosed MCL patients diagnosed by the Department of Pathology of our hospital between January 1, 2011, and May 30, 2016, were retrospectively analyzed starting from November 15, 2016. This study was approved by the Ethics Committee of our institution. All patients signed informed consent forms of admission, laboratory examinations, and treatment during hospitalization. The immunohistochemistry results were positive for Cyclin D1 in the tissue biopsies of all enrolled patients in this study. MCL was confirmed by at least 2 lymphoma pathologists in our hospital. Only 16 patients with bone marrow involvement received fluorescence in situ hybridization (FISH) detection of bone marrow cells. 13 cases showed t (11;14)(q13;q32) positive and 3 cases negative. The specific collected data included gender, age, clinical symptoms at intitial diagnosis, involved sites, Eastern Cooperative Oncology Group (ECOG) score, laboratory tests (including blood tests and bone marrow examination), imaging results, Ki-67 expression in tumor cells from pathological tissues. Accordingly, we reconfirmed the Ann Arbor stage, International Prognostic Index (IPI), MIPI, and simplified MIPI(s-MIPI) of the patients.^[6,7]^ Additionally, treatment regimens, efficacy evaluation, and disease outcomes of the patients were also collected.

### 2.2 Treatment regimens

Among 83 patients in this study, 3 patients refused treatment after diagnosis, and 5 patients were enrolled in a clinical trial; therefore, these patients were excluded from the efficacy and survival assessments. The initial induction therapy of 75 patients were as follows: 17 cases in the cyclophosphamide, doxorubicin, vincristine, and prednisone (CHOP) regimen group, including 1 case of the cyclophosphamide, vincristine, and prednisone (COP) regimen, 1 case of the attenuated CHOP (miniCHOP) regimen, and 1 case of the CHOP + etoposide(CHOPE) regimen; 37 patients in the rituximab + CHOP (RCHOP) regimen group, including 1 case of the rituximab + COP (RCOP) regimen. Additionally, 6 cases received cyclophosphamide, doxorubicin, vincristine, and dexamethasone, alternated with high-dose methotrexate and cytarabine (HyperCVAD/MA) regimen, and 15 cases received the HyperCVAD/MA regimen combined with rituximab (RHyperCVAD/MA). The salvage treatment regimens for relapsed patients included the mesna, ifosfamide, mitoxantrone, and etoposide (MINE) regimen, the gemcitabine, dexamethasone, and cisplatin (GDP) regimen, the rituximab, dexamethasone, high-dose cytarabine, and cisplatin (RDHAP) regimen, and the gemcitabine + oxaliplatin (GEMOX) regimen. Some patients received regimens containing lenalidomide and bortezomib.

### 2.3 Standards of the efficacy evaluation

The efficacy evaluation was performed for patients who finished at least 2 courses of therapy. According to the standardized response assessment by the National Cancer Institute-sponsored, international working group in 1999,^[8]^ treatment response was divided into complete remission(CR), complete remission unconfirmed (CRu), partial remission(PR), stable disease(SD), and progressive disease(PD).

### 2.4 Follow-up

All patients were followed up by telephone interview. The last follow-up date was November 30, 2016.

### 2.5 Statistical analysis

The statistical analyses were performed using SPSS 22.0. The measurement data were expressed as the median and range and the enumeration data as percentages. Comparisons of clinical characteristics and treatment efficacy of the patients were performed using the Chi-square test. The effects of the clinical and laboratory characteristics on patients’ survival were analyzed using the Kaplan–Meier method. Log-rank method was used to compare the effects of different factors on the disease prognosis. Multivariate COX regression analysis was performed to investigate the independent prognostic factors which derived from factors with statistical significance (*P* <.05) in the univariate analyses. *P* <.05 was considered as significant difference.

The progression-free survival (PFS) of patients referred to the time period from the time of initial treatment to disease progression or patient's death of any cause; for patients who were still in remission at the last follow-up, the last follow-up date was seen as the end point. The OS referred to the time period between the time of initial treatment to patient's death of any cause; for patients who were still alive at the last follow-up, the last follow-up date was used as the end point.

## 3 Results

### 3.1 Clinical characteristics

This study enrolled 83 newly-diagnosed MCL patients whose diagnosis were confirmed by the Department of Pathology of our hospital. During the same timeframe (5 years and 5 months), a total of 1336 cases of NHL were newly diagnosed in our hospital; therefore, MCL accounted for approximately 6.2% of the NHL cases. Of 83 MCL patients, 73 patients were male and 10 were female. The male to female ratio was 7.3:1; which was well higher than the ratio of 2.9:1 reported in European and American countries (*P* = .005).^[7]^ The median age was 59 (7–84) years, and 25 patients (30.1%) were above 65 years of age.

A total of 61 patients (73.5%) had extranodal organ involvement, among which bone marrow involvement was the most common with 36 cases (43.4%), followed by gastrointestinal involvement with 24 cases (28.9%). Other involved sites included the liver, kidney, lung, nasopharynx, parotid gland, and thyroid gland. 48 patients (57.8%) had single extranodal organ or site involvement, 13 cases (15.7%) had 2 or more extranodal organs involvement. Massive lesion with a diameter longer than 10 cm was seen in 5 cases (6.0%), and 21 patients (25.3%) had lesions with a diameter longer than 3 cm.

According to Ki-67 expression in the tumor cells from the pathological tissues of the patients in this study, 50.6% (40/79) cases were found to be lower than 30%, whereas 39 cases (49.4%) were higher than 30%. Clinical assessment showed that 1 case (1.2%) was at stage IIA according to Ann Arbor staging, 18 cases (21.7%) at stage IIIA, 2 cases (2.4%) at stage IIIB, 38 cases (45.8%) at stage IVA, and 24 cases (28.9%) at stage IVB. The percentage of patients at stage IV was significantly lower than that in European and American patients^[7]^ (74.7% vs 87.4%, *P* = .001). The IPI score showed that 25 patients (30.1%) were in the low-risk group, 38 patients (45.8%) in the low-intermediate-risk group, 17 patients (20.5%) in the high-intermediate-risk group, and only 3 patients (3.6%) in the high-risk group. Overall, there were only 20 patients (24.1%) in high-intermediate-risk and high-risk groups (IPI score 3 points and above). The median MIPI score was 6.13 (4.67–9.47). The s-MIPI scoring results demonstrated that 38 cases (45.8%) were in the low-risk group, 26 cases (31.3%) in the intermediate-risk group, and 19 cases (22.9%) in the high-risk group. Compared to European and American patients, there were significantly more low-risk patients in this study (*P* = .035).^[7]^ Additionally, this study showed that the majority of female patients (60.0%) were low-risk, whereas 43.8% of male patients were low-risk patients.

### 3.2 Efficacy analysis

Among the 75 patients who had received treatment in our institution, patients received chemotherapy with a median of 6 courses (range 2–9 courses). The age, ECOG score, Ann Arbor staging, MIPI score, and number of chemotherapy courses did not significantly differ among the 4 treatment groups. The overall response rate (ORR) of the regimen groups containing rituximab was significantly higher than those without rituximab (88.5% vs 65.2%, *P* = .021). Of these groups, the ORR in the RCHOP group was significantly superior than the CHOP group (*P* = .035), whereas there was no statistical difference in ORR between the RHyperCVAD/MA group and the HyperCVAD/MA group (*P* = .500). Besides, the percentage of CR and CRu of the groups containing rituximab were also significantly higher than those without rituximab (53.8% vs 13.0%, *P* = .001). Of these regimens, CR and CRu were significantly higher in the RCHOP group and the RHyperCVAD group than in the CHOP group and the HyperCVAD (*P* = .006 and .033, respectively). Regardless of the ORR or CR + CRu, no statistical difference was found between CHOP group and HyperCVAD/MA group or between RCHOP group and RHyperCVAD/MA group (Table 1).

**Table 1:**
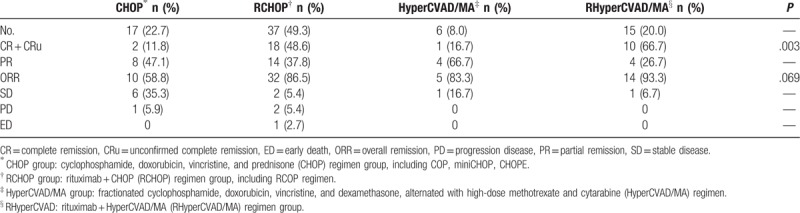
Therapeutic effect of the 4 treatment groups.

### 3.3 Survival analysis

Follow-up was performed on 75 patients who underwent treatment in our institution. Three patients were lost to follow-up. The median follow-up time was 19.1 m (range: 0.7–71.3 m). At the end of follow-up, there were 30 cases of death, and the overall mortality was 40.0%; of these cases, 25 patients (33.3%) died of disease progression, and 5 patients (6.7%) died of treatment-related causes. Relatively higher treatment-related mortality was seen in patients who received the HyperCVAD ± R regimens (37.5% vs 9.1%, *P* = .081). The expected median PFS of the patients was 27.8 m (95% CI: 11.3–44.3 m), and the 1-year and 3-year PFS rates were 77.2% and 46.9%, respectively. The expected median OS was 58.1 m (95% CI: 31.2–85.0 m), and the 1-year and 3-year OS rates were 81.0% and 60.1%, respectively.

**Figure 1:**
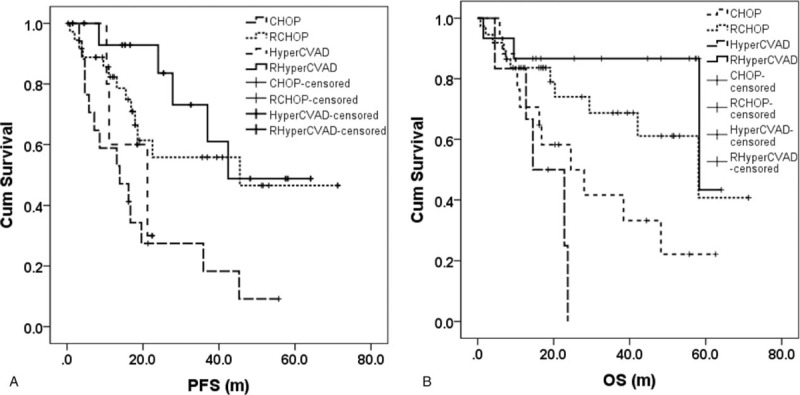
Patients’ survival of the 4 treatment groups. The differences in PFS and OS among the 4 treatment groups were significant (*P* = .005 and .003, respectively). OS = overall survival, PFS = progression free survival.

The median PFS of the patients in the CHOP group, the RCHOP group, the HyperCVAD/MA group, and the RHyperCVAD/MA group were 13.9 m, 45.5 m, 21.2 m, and 42.4 m, respectively. The differences in PFS among the 4 groups were significant (*P* = .005) (Fig. 1A). But no difference was shown between CHOP and HyperCVAD (*P* = .474) or between RCHOP and RHyperCVAD (*P* = .394). The median OS of the above groups was 24.5 m, 58.1 m, 14.5 m, and 58.3 m, respectively, and the differences were significant (*P* = .003) (Fig. 1 B). Still, no difference was observed between CHOP and HyperCVAD (P = .140) or between RCHOP and RHyperCVAD (*P* = .260). Both the PFS (45.5 m vs 16.2 m, *P* = .001) and OS (58.3 m vs 22.8 m, *P* = .001) were significantly longer for the treatment regimens containing rituximab than for those without rituximab.

### 3.4 Effects of the clinical characteristics on patients’ survival

In this study, clinical characteristics including gender (female), peripheral white blood cell count <10 × 10^9^/L, platelet count ≥130 × 10^9^/L, monocyte count <0.5 × 10^9^/L, normal serum β2 microglobulin level, normal albumin level, normal hemoglobin level, normal C-reactive protein level suggested significantly better PFS (*P* < .05). Besides, patients with normal lactate dehydrogenase (LDH) concentration showed slightly better PFS (*P* = .064). Patients with age <65 years, Ki-67-positive rate ≤30%, peripheral white blood cell count <10 × 10^9^/L, platelet count ≥130 × 10^9^/L, monocyte count <0.5 × 10^9^/L, normal serum β2 microglobulin level, normal albumin level, normal C-reactive protein level had better OS (*P* <.05). Normal LDH concentration and female also indicated longer OS (*P* = .078). Patients at stage IV or with B symptoms had worse PFS and OS. s-MIPI, rather than MIPI, was significantly associated with PFS (*P* = .008) and OS (*P* <.001) of the patients. This study indicated no significant effects of the mass size and the IPI scoring system on the PFS and OS of the patients. Details are shown in Table 2.

**Table 2:**
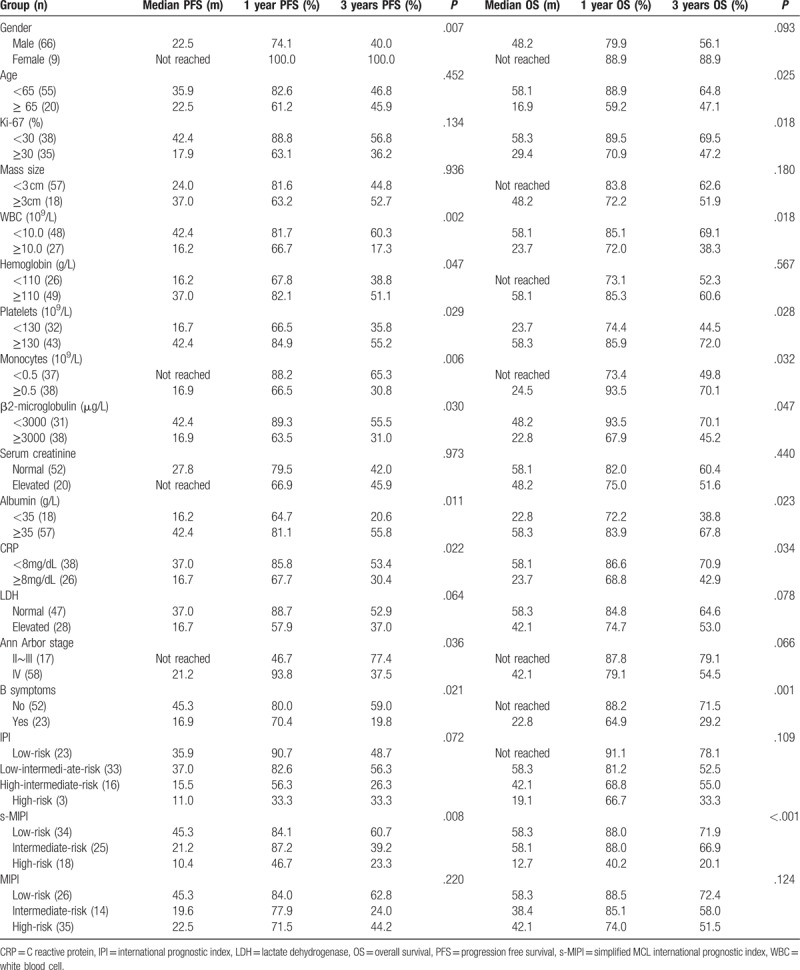
The impact of clinical characteristics on patients’ survival.

### 3.5 Multivariate COX regression analysis

According to the univariate analysis results, the age, gender, B symptoms, white blood cell count, monocyte count, hemoglobin level, platelet count, serum β2 microglobulin level, albumin level, C reactive protein level, Ki-67, Ann Arbor stage, s-MIPI score, and treatment regimens of the patients were separately included in the COX regression analyses of patients’ PFS and OS. The results suggested that B symptoms, the s-MIPI score, and administration of immunochemotherapy were independent factors associated with PFS and OS of MCL patients (Table 3).

**Table 3:**
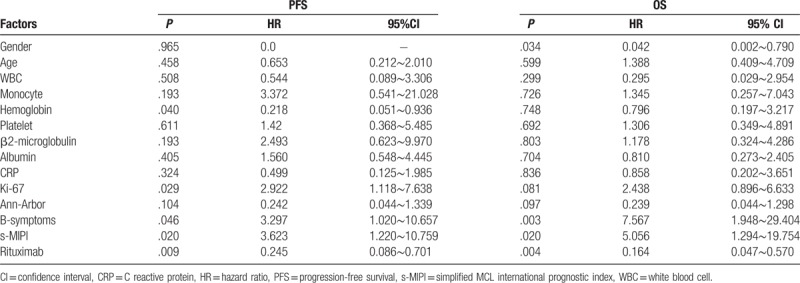
Multivariate COX regression analysis.

s-MIPI stratification and the presence of B symptom were combined to create s-MIPI-B stratification method which classified patients as low-risk, middle-risk, and high-risk groups. With this method, patients in low-risk group of s-MIPI without B symptom were defined as low-risk, patients in high-risk group of s-MIPI and patients in low-risk group of s-MIPI with B symptom as high-risk, the rest as middle-risk. Median PFS of the 3 groups were not reached (NR) vs 35.9m (95% CI 13.0–58.8) vs 13.1m (95% CI 7.2–19.0) (*P* = .001), median OS NR vs 42.1m (95% CI 27.8–56.4) vs 16.8m (95% CI 9.6–24.0) (*P* <.001). 3-year PFS was 74.9%, 43.4% and 16.1%, respectively (*P* = .001). 3-year OS were 84.4%, 62.2%, 27.6% (*P* <.001) (Fig. 2).

**Figure 2:**
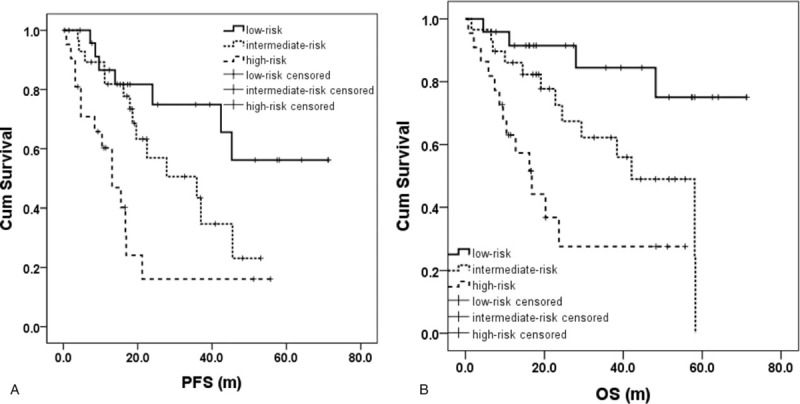
Patients’ survival of the 3 risk groups. By s-MIPI-B stratification method, the PFS and OS of patients in 3 risk groups were significantly different. OS = overall survival, PFS = progression free survival, s-MIPI = simplified MCL international prognostic index.

## 4 Discussion

MCL is a group of B cell lymphoma with distinct clinicopathological characteristics that is highly prevalent among elderly males. Tumor cells appear as CD5+ mature small lymphocytes, of which a small portion present as the blastic type. The majority of patients are at Ann Arbor stage III-IV at diagnosis.^[3,4,7]^ This study showed that the percentage of male patients among Chinese MCL patients was significantly higher than that among European patients. The percentage of patients at Ann Arbor stage IV and evaluated as high risk by s-MIPI at diagnosis was lower. However, the different characteristics between Chinese and European patients need to be further confirmed. Extranodal involvement of MCL disease is very common, especially gastrointestinal involvement.^[9–11]^ 29% of all patients in our study were accompanied with gastrointestinal involvement, in accordance with literatures.^[10,11]^ Nevertheless, the true incidence of gastrointestinal involvement in MCL patients might be much higher than the incidence observed in the clinic.^[10,11]^ The positive rate of gastrointestinal involvement by upper and/or lower gastrointestinal endoscopy and tissue biopsy can exceed 90%. Romaguera et al concluded that gastrointestinal endoscopy resulted in changes in clinical management in only 4% patients.^[10]^ However, we believe gastrointestinal endoscopy should still be recommended for newly diagnosed MCL patients and as assessment of complete response after therapy.

Similar to other B cell lymphomas, immunochemotherapy that combines chemotherapy and rituximab targeting cell surface CD20 can significantly improve the efficacy and prognosis of patients.^[3,12,13]^ Lenz et al^[3,12,13]^ compared the efficacy between the RCHOP and CHOP regimens as first-line therapy of MCL in a prospective, randomized, controlled trial. The results showed that the RCHOP regimen had a higher treatment ORR (94% vs 75%, *P* <.001) and CR (34% vs 7%, *P* <.001) than the CHOP regimen. The retrospective study of Dreyling et al^[13]^ on 652 cases of MCL patients demonstated that the OS of patients who received the first-line immunochemotherapy was significantly better than the OS of those who received simple chemotherapy (37 m vs 25 m, *P* <.001). The results in this study also suggested that both ORR and prognosis were significantly better for the patients treated with immunochemotherapy than for those with simple chemotherapy. Recent clinical studies showed that the introduction of high-dose chemotherapy to young patients with basically normal functions of important organs could further optimize their prognose.^[4,12,14,15]^ For example, the ORR and CR of treatment using the combination of HyperCVAD/MA containing large doses of MTX and cytarabine and RHyperCVAD containing rituximab were 97% and 87%, respectively,^[14]^ and the median OS was not achieved after 10 years of follow-up. In our study, the effective rate in the RHperCVAD/MA group reached 93.3%, which was similar to literature reports. PFS and OS were both significantly better in the RHyperCVAD group than in the HyperCVAD group, suggesting that the application of rituximab further improved the efficacy and prognosis even after the administration of high-dose chemotherapy.^[15]^ However, PFS and OS were not obviously different between CHOP and HyperCVAD group, or between RCHOP and RHyperCVAD group, suggesting that patients may not benefit from high dose chemo. The OS of patients aged over 65 years on the HyperCVAD-like regimen was significantly shortened and possibly associated with severe treatment-related adverse reactions.^[15]^ Furthermore, regimens based on high-dose cytarabine, such as the DHAP regimen, accompanied with subsequent ASCT significantly improved the prognosis of patients aged 65 years or younger.^[4]^ For elderly patients, bendamustine combined with rituximab could obtain unexpected efficacy. Novel targeted drugs, such as bortezomib, lenalidomide, and ibrutinib are all excellent choices for the treatment of elderly MCL patient.^[12,16–21]^

MCL is a group of NHL subtypes with invasiveness and high heterogeneity which shows relatively poor prognosis. Some patients present an indolent process and long-term stability, whereas other patients experience rapid progression and very short survival. Therefore, personalized treatment for MCL patients is recommended based on prognostic risk stratification. Structural overexpression of CCND1 in MCL tumor cells is a driving cytogenetic abnormality in MCL tumorogenesis.^[1]^ However, given that CCND1 is a weak oncogene, the aggressive disease pattern always results from secondary cytogenetic events. Besides, abnormal expression of neuronal transcription factor SOX11 can be observed in classic invasive MCL, which was deemed to indicate inferior outcome. Recently, the prognostic effect of SOX11 appeared conflicting.^[22–25]^ Thus, 2017 International Conference on Malignant Lymphoma in Lugano brought up that SOX11 may not have prognostic value.^[26]^ However, the technique used in chromosome and genetic testing is more complex and high-cost, prognostic stratification based on the clinical characteristics may be more economical and convenient. In 2008, based on the age, physical status, serum LDH level, and white blood cell count of the patients, Hoster et al established the MIPI scoring system, which has been proved highly predictive of the prognosis of MCL patients and is significantly superior to the IPI scoring system that is commonly used for NHL.^[6,7]^ The factors in this system are classified to form the s-MIPI for clinical use. This study also showed that the s-MIPI score was an independent prognostic factor affecting the PFS and OS of the patients which significantly indicated 3-year OS of 20.1% in high-risk patients. The 3-year OS rates in the low-risk and intermediate-risk groups were 71.9% and 66.9%, respectively, with no significant difference between these 2 groups. Moreover, we found that the MIPI in this study did not have a significant risk stratification function, which was similar to the multi-center and retrospective data from 1 Japanese study.^[27]^ As proposed by Hoster et al,^[7]^ the s-MIPI was more appropriate than the MIPI in the general population in clinical practice.

The study suggested that Ki-67 was closely associated with the survival of MCL patients. A high Ki-67 index suggested poorer PFS and OS.^[28–30]^ The MIPI-c prognostic system, consisted of the Ki-67 index and s-MIPI, showed better prognostic effects.^[28]^ In this study, a higher Ki-67 index suggested poorer OS (median survival 58.3 m vs 29.4 m) but was not obviously predictive of PFS. The treatment regimens and the number of treatment courses of the patients exhibited larger variations probably owing to this study being retrospective. According to other literatures, factors that could predict the prognosis of MCL patients also included the gender, age, B symptoms, spleen involvement, bone marrow involvement, tumor diameter, peripheral white blood cell count, monocyte count, hemoglobin, β2 microglobulin, and albumin level.^[27,31–38]^ In this study, we also found that B symptoms, monocyte count, serum macroglobulin level, and albumin level had significant effects on the OS of patients. The B symptoms, in particular, turned out to be an independent prognostic factor associated with the PFS and OS of the patients, which was consistent with literature reports.^[6,27]^ Notably, in this study, the efficacy and outcome of female patients were both significantly better than those of male patients. None of the female patients experienced relapse during treatment and follow-up, and only 1 female patient died of severe infection after treatment. Whether this result suggests that female patients can adopt milder treatment regimens requires further investigation.

Additionally, we found that platelet count <130 × 10^9^/L and C-reactive protein concentration ≥8 mg/dL were closely associated with shorter OS of the patients. This result has not been proposed among MCL patients. The platelet count and serum C-reactive protein level at diagnosis has been reported to be independent influencing factors of PFS and OS in patients with diffuse large B-cell lymphoma (DLBCL).^[39–43]^ Among the prognostic factors for MCL, another factor in addition to Ki-67 that was closely associated with the biological characteristics of the tumor cells was high SOX11 expression. Abnormal cell and molecular genetics, such as TP53 mutations, were closely associated with the MCL prognosis.^[28–30,44–47]^ Because the MIPI scoring system does not include the biological characteristics of tumor cells and seemingly cannot classificate low-risk and intermediate-risk groups in Asian populations, further establishment of a better MCL prognostic system in Asian populations should be taken into consideration.

## 5 Conclusion

Among the Chinese population, MCL was more common in males. However, the proportion of late-stage and high-risk patients were significantly lower than that of European patients. Immunochemotherapy significantly improved the prognosis of MCL patients. Multivariate analysis demonstrated that B symptoms, s-MIPI, and administration of rituximab were independent clinical prognostic factors for the patients.

## Author contributions

**Conceptualization:** Jing-Song He, Zhen Cai.

**Formal analysis:** Xi Chen, Ji-Min Shi.

**Funding acquisition:** He Huang, Zhen Cai.

**Investigation:** Ji-Min Shi, Gao-Feng Zheng.

**Methodology:** Wei-Yan Zheng, Wen-Jun Wu.

**Resources:** Guo-Qing Wei, Jie Sun, Yi Zhao, Gao-Feng Zheng, He Huang.

**Software:** Yi Zhao.

**Supervision:** Guo-Qing Wei.

**Validation:** Jie Sun.

**Visualization:** Wei-Yan Zheng, Wen-Jun Wu.

**Writing – original draft:** Xi Chen.

**Writing – review & editing:** Jing-Song He.
